# Emerging Single-Cell Technological Approaches to Investigate Chromatin Dynamics and Centromere Regulation in Human Health and Disease

**DOI:** 10.3390/ijms22168809

**Published:** 2021-08-16

**Authors:** Laura Leo, Nunzia Colonna Romano

**Affiliations:** Dipartimento di Biologia e Biotecnologie “Charles Darwin”, Istituto Pasteur Italia, Università Sapienza, 00185 Rome, Italy

**Keywords:** epigenetics, centromere, chromatin regulation, single-cell epigenetics, emerging technologies, epigenetics in human health & disease

## Abstract

Epigenetic regulators play a crucial role in establishing and maintaining gene expression states. To date, the main efforts to study cellular heterogeneity have focused on elucidating the variable nature of the chromatin landscape. Specific chromatin organisation is fundamental for normal organogenesis and developmental homeostasis and can be affected by different environmental factors. The latter can lead to detrimental alterations in gene transcription, as well as pathological conditions such as cancer. Epigenetic marks regulate the transcriptional output of cells. Centromeres are chromosome structures that are epigenetically regulated and are crucial for accurate segregation. The advent of single-cell epigenetic profiling has provided finer analytical resolution, exposing the intrinsic peculiarities of different cells within an apparently homogenous population. In this review, we discuss recent advances in methodologies applied to epigenetics, such as CUT&RUN and CUT&TAG. Then, we compare standard and emerging single-cell techniques and their relevance for investigating human diseases. Finally, we describe emerging methodologies that investigate centromeric chromatin specification and neocentromere formation.

## 1. Introduction

In 1942, for the first time, Waddington coined the term ‘epigenetic’ as the branch of biology that studies the causal interactions between genes and their cellular products and implements the phenotype [1].

In past decades, the concept of epigenetic regulation has evolved, thanks to technological advances that have revolutionised the investigation of biological phenomena. Currently, epigenetics is defined as the science that studies stable and potentially heritable changes in gene expression and the phenotype occurring without alterations in the DNA sequence [2].

**Figure 1:**
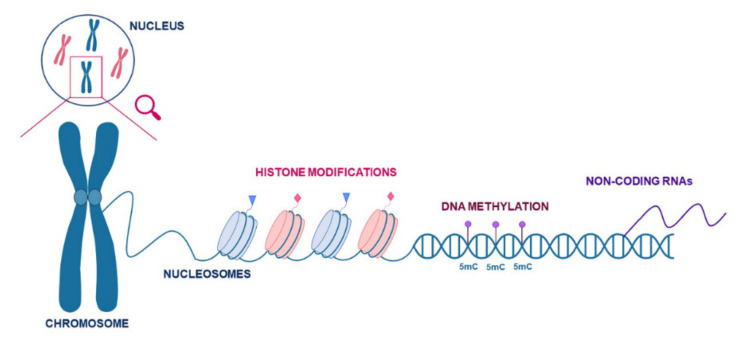
Schematic representation of the main epigenetic mechanisms: DNA methylation, histone post-translational modifications (PMTs) and non-coding RNAs. In the nucleus, chromatin is organised in peculiar chromosome structures composed of DNA wrapped around nucleosomes. Nucleosomes are formed by four histones that can be modified in their *N*-terminal tails to open or compact chromatin, for example, acetylation, phosphorylation and methylation. DNA methylation usually occurs at 5′-position cytosine residues on CpG islands. Non-coding RNAs can be transcribed but not translated in a protein playing a crucial role in epigenetic regulation of the genome. All epigenetic modifications work together to determine an epigenetic code to regulate chromatin organisation and gene expression.

The main epigenetic mechanisms include DNA methylation [3], histone modifications [4] and non-coding RNAs (ncRNAs) [5] (Figure 1). These mechanisms are dynamically regulated in response to developmental and environmental stimuli; thus, they establish feedback in the control of several biological processes such as gene expression, genome architecture, growth, apoptosis, alternative splicing, DNA repair and ultimately, evolution [6,7,8].

Most often, DNA methylation is found in association with silent chromatin states and typically takes the form of methylation of cytosine; such a modification mainly occurs on CpG islands, which are typically found in regulatory regions such as enhancers and promoters of genes or in repetitive DNA sequences [9].

Histone modifications (methylation, acetylation, phosphorylation, ubiquitination and many others) are key regulatory modifications resulting in changes in transcription, DNA replication and chromosome condensation [6,10,11]. Generally, acetylation is associated with euchromatin, a general term for transcriptionally active chromatin states [12], while methylation may be found in both euchromatin or heterochromatin (transcriptionally inactive) regions depending on the specific lysine/arginine residues that are modified [7].

Non-coding RNAs (ncRNAs) are a heterogeneous class of regulatory molecules derived from genes that are transcribed but not translated into proteins. They can be further classified in small-interfering RNAs (siRNAs), microRNAs (miRNAs), Piwi-interacting RNAs (piRNAs) and long non-coding RNAs (lncRNAs) which have key roles in many biological processes [13,14].

Epigenetic modifications are continuously and dynamically modulated, especially by environmental factors. Indeed, the main benefit they provide lies in the ability to ‘fine-tune’ gene expression in line with progressive ontogenic and environmental changes. Thus, epigenetic markers are preferred developmental indicators [15,16,17], being more stable, accurate and specific than transcriptional markers [18].

Human health is impacted by epigenetics, and disruption in the correct balance of open and closed chromatin states can result in the onset of epigenetic machinery disorders. Growing evidence support that epigenetic machinery may profoundly affect human health.

Mendelian disorders of the epigenetic machinery (MDEMs) are genetic diseases caused by mutations in genes coding for epigenetic factors and are often associated with intellectual disability, revealing that epigenetic mechanisms are very crucial for normal neurological development [19].

In addition to monogenic diseases, epigenetic mechanisms are significantly involved in multifactorial diseases such as neurodegenerative disorders [20], metabolic dysfunction [21] and cancer progression [22], although it is unclear the degree by which epigenetic alterations are the cause or the consequence of pathogenesis. For example, epigenetic variability between cancer cells that may arise in response to environmental stimuli may exacerbate cell-to-cell heterogeneity and contribute to cancer progression and resistance to therapy [23]. For many years, cell variability has been hard to quantify due to the lack of specific methodologies. For instance, chromatin immunoprecipitation followed by sequencing (ChIP-seq) is a major technique to profile chromatin accessibility and DNA–protein interactions in a population of cells but is not sufficiently sensitive to quantify epigenetic features at the level of single cells. In recent years, single-cell sequencing techniques have been developed and applied to the study of epigenetics. For instance, Bolhaqueiro and colleagues have developed a methodology to analyse single-cell DNA sequencing data to reveal genome changes and chromosomal aberrations, such as duplication or deletions, to explore cell-to-cell variability in a heterogeneous cancer cell population [24].

Chromosomal instability (CIN) is a hallmark of many types of cancer. However, how CIN contributes to cancer progression remains to be elucidated. CIN is characterised by loss or rearrangement of genetic material during cell division, resulting in aneuploidy, chromosome structural alterations and segregation errors. Thus, CIN can increase genetic heterogeneity between tumour cells. A key structure for chromosome segregation is a chromatin region called the ‘centromere’. Centromeres recruit the kinetochore complex, a proteinaceous structure that provides the physical attachment to the microtubules of the mitotic spindle. Any alteration in the architecture of the centromere can result in cell cycle defects, leading to chromosomal instability. Indeed, the overexpression of CENP-A, the main epigenetic centromere marker, has been reported in several types of tumours, and it correlates with poor prognosis for patients. The molecular consequences of CENP-A overexpression in cancer cells are still unclear [25,26,27]. However, CENP-A overexpression drives chromosomal instability and aneuploidy, as shown in a cancer cell line and in a xenograft mouse model [28].

Epigenetic marks can affect the cells’ transcriptional output, and consequently, their function. Therefore, understanding their effects on single-cell resolution can determine variations that occur in cell fate and function. Owing to this need, new techniques for studying single-cell epigenomics have been developed and previously deepened in some respects [29,30,31,32,33,34]. In summary, although epigenetic profiling of cell populations has contributed new insights on the role that epigenetic marks have on cellular function, understanding the effects of these marks at single-cell resolution is needed to appreciate cell-to-cell variability in fate determination and function in health and disease [30,31,32,33].

In this review, we discuss single-cell methodologies that have recently emerged and that may provide new impetus in achieving the goals of ‘personalised medicine’. We present a critical review of such techniques examining strengths, challenges and limitations and offering insight in the epigenetic field into molecular components of the genome and its functional output.

## 2. Single-Cell Sequencing and Common Applications

Over the last few years, single-cell sequencing (SCS) has emerged as a powerful set of technologies applied to a multitude of biological questions. Before the widespread adoption of single-cell genomics, RNA (in the form of cDNA) or (genomic) DNA sequencing was performed on nucleic acids isolated from whole tissue. Tissues are made of different cell types that are difficult to separate. Moreover, even within a single cell type, the population is heterogeneous, which in large part is due to the stochastic accumulation of mutations introduced during DNA replication [35]. In this regard, physiological functions in health and disease are distinguished by the interplay between cells, but traditional sequencing technologies afford low resolution to the problem. Conversely, SCS can detect heterogeneity among individual cells and can lead to the discovery of new cell types (or sub-types), rebuilding the cell development trajectory. Generally, SCS is used to analyse gene expression, to identify events of sister chromatin exchange and to define the methylation status of genes of interest. [36]. For instance, SCS applied on post-mortem brain tissue has allowed studying the cellular composition of some isolated neurons and their involvement in some neurodegenerative diseases or brain lesions [37]. SCS technologies have been applied in stem, neuronal or glial cells to study the epigenetic profiles of open chromatin and investigate its role in the pathogenesis of diseases. In combination with single-cell immune profiling, SCS also allows obtaining immune profiling with the possibility to characterise new immune cell types and states. From this point of view, SCS can aid in identifying new drug targets and verify whether they work as expected.

## 3. Single-Cell Technology Approaches to the Study of Neurodegenerative, Metabolic and Multifactorial Diseases

In light of the potential impact on several diseases’ development, epigenetics is a powerful field to study all mechanisms and risk factors contributing to their progression. Indeed, in an organism, the epigenome is normally established at the end of embryonic development and subsequently subjected to several modifications, which in not common cases, can unbalance the activation state of a gene, resulting in the onset of cancer [38]. Even if the functional relationship between epigenomic and transcriptional dysregulations and neurodegenerative and other age-linked disorders is not clear, the introduction of single-cell sequencing technology has certainly provided more insights about gene-expression differences that originate from specific cell types. Several evidences show that DNA methylation and histone post-translational modifications could play a pivotal function in Alzheimer’s disease (AD), in which RNA-seq analysis reveals widespread transcriptomic dysregulation with altered splicing [39]; in ageing-associated phenotype [40], as demonstrated in podocytes of aged mice, in which RNA-seq analysis showed differentially expressed genes to distinct targeting of the ageing process; and in Atherosclerosis, where several genome-wide epigenomic mapping techniques have been performed in a low set of cells [41,42], as also confirmed by the epigenetic alterations in auto-immune disorders [43]. Recently, Mathys et al. demonstrated the key role of glial–neuronal interactions in response to AD pathology thanks to a single-cell transcriptomic analysis through which important myelinisation regulators, such as LINGO1, have been identified as alternating between neuronal and glial cells, suggesting their high therapeutic potential [44]. Additionally, in AD, reference maps of genome-wide distribution of DNA methylation state were created to characterise epigenetic alterations during the disease progression [45]. General deregulation of epigenetic machinery has also been shown in AD with a histone-deacetylation alteration [46], a ‘global’ DNA methylation and hydroxy-methylation profile observed in the patients [47], that along with ncRNA-associated gene silencing, are taking on an increasingly relevant role as biomarkers and therapeutic targets for AD [48]. In this regard, Garcidueñas et al. demonstrated a general decrease of the H3K9me2 and H3K9me3 (H3K9me2/me3) both in AD patients with higher levels of the DNA double-strand-break marker γ-H2AX in young adults who resided in metropolitan versus low-pollution areas, suggesting the important relationship between epigenetic gene regulation and environmental exposure [49]. Environmental factors and ageing have been included in important human-induced pluripotent stem cell (iPSC)-based models for the recapitulation of cytopathic phenotypes and drug screening of many neurodegenerative diseases, in which single-cell technologies play a significant role [50]. Indeed, epigenetic changes have assumed a prominent value as biomarkers in the treatment not only of neurodegenerative diseases but also in the normal development of the brain in response to different environmental stimuli [51]. In this context, the role as biomarkers of the Transposable elements (TE) and the epigenetic marks associated with them has also been highlighted in order to understand the tissue or cell-specific epigenomic impact of environmental exposures and related diseases [52].

In recent years, more attention has been paid to the phenomenon of cellular heterogeneity in which metabolism can play a role. The complexity of the mechanisms underlying the functioning of the metabolome, both in pathological and non-pathological conditions, is a topic of high interest to which transcriptomic and proteomics single-cell analysis have contributed enormously [53]. In this regard, there are several correlations between metabolic disorders and recent technological improvements in single-cell and omics epigenetic profiling that shed light on new opportunities for research and therapy discovery. Furthermore, the transcriptomic profiling by single-cell RNA sequencing (scRNA-seq) has helped to understand important gene expression dynamics during the different stages of development in mouse and human germ cells [54,55,56], mouse liver development [57] and human fetal kidney growth [58].

Additionally, in cancer cells, many global epigenetic alterations have been observed, suggesting that epigenetic aberrations play a key role in cancer initiation and progression. In order to ensure effective diagnosis and treatment for cancer, scRNA-seq helps in the detection of genetic information, in the control of genes and in the difference between gene expressions in individual cells. Recently, Park et al. using scRNA-seq, revealed that the major contribution in pathogenicity in several mouse kidney diseases could be found in cell-type-specific markers, conversely to that which has previously been shown by transcriptomic studies studying the global influence of different cell groups [59].

It has also been shown that obesity and the alteration of genes related to fat metabolism are important negative regulators of histone methylation and autophagic processes, as demonstrated by Pan et al. with single-cell RNA transcriptome sequencing in mouse embryos [60]. Additionally, this approach further revealed how specific inflammatory-related genes are also altered in the oocytes of obese women compared to those with normal body weight [61], suggesting the useful contribution of scRNA-seq in the exploration of complex biological systems such as the immune system.

Of note, studies in *Drosophila melanogaster* have significantly contributed to deepening our knowledge of human diseases, unlocking molecular pathogenesis pathways in different fields, such as neurodegenerative disease, cardiovascular, metabolic and immunological disorders [62]. In an interesting study, the expression of genes linked to several important metabolic pathways has also been investigated in *Drosophila* eye-disc development process, in which the authors have identified an altered glycolytic activity in Rbf (retinoblastoma family protein) mutant cells compared to wild-type, using a scRNA-seq study [63].

*Drosophila*’s blood system has also been used as a useful tool for identifying new cell types that have not yet been discovered and potentially expressed even in the human immune system, acknowledging the analysis of single subtypes of cellular populations that with a common sequencing would not be recognisable [64].

In this regard, considering the importance as common epigenetic markers of miRNAs detection, histone variants and PMTs, and also histone detection in patient’s blood, could be a non-invasive system to research epigenetic markers that distinguish health from pathological conditions. Furthermore, in the last decade, new technological approaches have been emerged to develop strategies to employ epigenetic biomarkers in diagnostics for human health. Moreover, epigenetic compound development could be advantageous to heal several disorders that display resistance to standard treatment, leading to solutions for biomedical problems.

Since diverse mutations can be encountered during the early cell divisions and sometimes cause genetic disorders and the onset of genetic variations, single-cell sequencing has allowed more clues on the cell type involved and on the disease progression. Indeed, single-cell genomics has made it possible to carry out wide-ranging studies of action in an organism’s life cycle, highlighting the effects of increased vulnerability during the early stages of development. The methods developed so far can allow the dissection of the single-cell genetic content in normal organs, pre-malignant states and established tumours, providing insights into the functioning of maintaining the genome in health and disease.

## 4. Chromatin Immunoprecipitation (Chip) as a General Method to Study Protein–DNA Interactions

**Figure 2:**
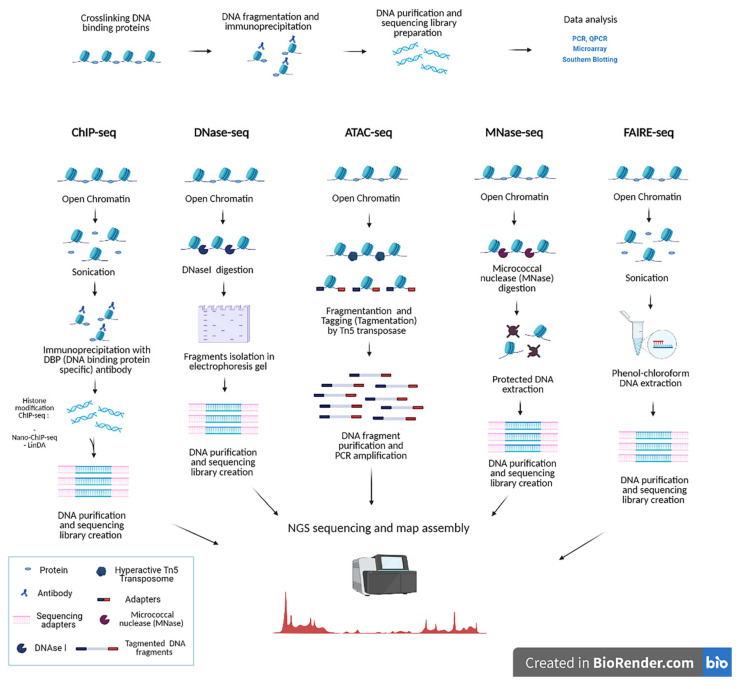
Overview of ChIP-seq and sequencing variants. ChIP-Seq combines chromatin immunoprecipitation with DNA sequencing to show specific transcription factor binding sites. DNase-seq reveals regions of open chromatin after DNase I endonuclease digestion as also in MNAse-seq, in which Micrococcal nuclease digests DNA to create a sequencing library. Additionally, ATAC-seq is used to study chromatin accessibility genome-wide in DNase I hypersensitive sites, in which an engineered Tn5 transposase cleaves DNA that is tagmented with primer DNA sequences to NGS sequencing. This method appears as complementary technique to DNAse-seq, MNase-seq and FAIRE-seq. In FAIRE-seq, used to detect the nucleosome-free regions of the genome, the chromatin is crosslinked with formaldehyde in vivo, sheared by sonication and phenol-chloroform extracted to NGS sequencing (created with biorender.com).

Cellular differentiation is a highly dynamic process that involves continuous interactions between DNA and proteins such as transcription factors, histones and histone-modifying enzymes. Years ago, a lack of information on the genomic distribution of histone modifications prevented the full understanding of the ‘histone code’. This refers to the instructive role conferred by specific histone modifications and their combinations to the structural organisation and expression of the genome [65]. Major advances were made possible by the development of approaches that combine protein-specific chromatin immunoprecipitation (ChIP) [66] with techniques for the identification of the associated DNA, such as PCR amplification of target sequences (ChIP-PCR) [67], DNA microarray hybridisation (ChIP-on-chip) and genome sequencing (ChIP-seq) [68,69,70] (Figure 2).

The aim of ChIP techniques is to identify which DNA sequences are bound to variants of structural or functional proteins in the chromatin. ChIP-PCR is limited to investigating candidate sequences, whereas the development of ChIP-on-chip has opened the door to unbiased genome-wide studies. However, ChIP-on-chip approaches suffer substantial technical limitations. A microarray chip is a solid substrate on which thousands of DNA sequences called ‘probes’ are arranged in rows and columns such that position reflects identity. Thus, the identification of the DNA sequences part of the immune-precipitated chromatin relies on their hybridisation to the probes after an amplification step [71]. Biases introduced during the amplification reaction, unspecific hybridisation signals and the need to probe several arrays to cover the entire human genome are the most noticeable limitations of this technique. An improvement was introduced with the advent of ChIP-SAGE (SAGE, serial analysis of gene expression) [72], useful to analyse histone modifications and DNA-binding proteins in entire unknown genomes. Generally, this method is based on short sequence tags, containing information about an mRNA and sequencing them to identify the abundance of a transcript in a cell. The ability to detect unknown genes is a useful advantage of ChIP-SAGE over microarray studies, making sets of experiments more comparable with each other and reducing background noise. However, the best results are obtained with ChIP-seq, currently the main method in epigenomic research [69,73,74]. Here, second-generation sequencing is used to identify the precipitated immune DNA, a very sensitive technique that is becoming progressively more affordable [75].

For many years ChIP-seq has been the method of choice for genome-wide mapping of protein–DNA interactions. However, some concerns have been raised recently [76,77]. In a typical chromatin immunoprecipitation reaction, first, the proteins are cross-linked to the DNA by chemical or physical means (such as formaldehyde or UV light). Then, the chromatin is precipitated using antibodies and fragmented by sonication or enzymatic activity. The DNA is extracted, modified (for example, by ligating ‘adapters’) and amplified to make libraries for sequencing. All these steps are vulnerable to bias. For example, cross-linking can cause epitope masking, leading to false negatives results [78]. Mechanical fragmentation by sonication can generate a heterogeneous pool of DNA fragments depending on the configuration of the chromatin and consequently sample-specific biases. Conversely, DNA fragmentation by enzymatic activity presents a risk because the enzyme could more efficiently cut some genomic regions (for instance, A/T rich) compared to others.

Recent advances on the traditional ChIP-seq method include the ChIP-exo assay that introduces a significant improvement in mapping protein–DNA interaction sites. It achieves up to a single base-pair resolution by employing an endo and exonuclease combination to cleave and remove all exposed DNA sequences but preserving those engaged in interactions with proteins [79]. Additionally, Nano-ChIP–seq [80] and single-tube linear DNA amplification (LinDA) assays have greatly improved sensitivity and require just a few hundred cells from which a few pg of DNA can be extracted (Figure 2). LinDA is also compatible with high-speed sequencing and an optimised T7 phage RNA polymerase linear amplification protocol because the whole reaction takes place in a single tube and with a single buffer [81]. Additionally, the ultra-low-input micrococcal nuclease-based native ChIP (ULI-NChIP) assay (Figure 2) can generate histone profiling from little starting material, for instance, from a low number of embryonic stem cells [82]. The primary constituents of chromatin, the nucleosomes, are organised at various levels of compaction that reflects different activation states. Several methods generate genome-wide profiles of nucleosome occupancy. Indexing-first chromatin immunoprecipitation (iChIP) works by barcoding total cellular chromatin [83]. Chromatin interaction analysis by Paired-End Tag sequencing (ChIA-PET) uses a Paired-End Tag (PET) strategy to detect long-range chromatin interactions mediated via a protein of interest [84]. This method aims to identify distal interactions between promoters and regulatory regions through tag mapping and identification of chromatin protein binding sites. Drop-ChIP and scChIP-seq analyse chromatin states in multiple single cells in parallel using molecular barcoding, combining droplet microfluidics with single-cell DNA barcoding technologies. Single cells are isolated into droplets containing lysis buffer and micrococcal nuclease (MNase). After, oligonucleotides-dependent cell-specific barcoding chromatin is introduced into the pooled droplets before immunoprecipitation to perform ChIP-seq [85]. ChIP-seq has also been incorporated in methods to study gene regulation, such as DNase-seq [86,87], FAIRE-seq [88] and ATAC-seq [89] (Figure 2). DNase-seq is used to identify nucleosome-free regulatory regions of the genome accessible to DNase I, an endonuclease of *E. coli*, which can only access DNA regions not wrapped around histones. After, lysis DNA fragments are generated by DNase I restriction and identified by Next Generation sequencing (NGS) [90]. FAIRE-Seq (Formaldehyde-Assisted Isolation of Regulatory Elements Sequencing) is based on a similar principle [88]. However, instead of permeabilising the cells, crosslinking takes place directly between DNA and genomic portions that are nucleosome-free (with open chromatin). After DNA fragmentation, DNA is extracted, amplified and analysed by NGS sequencing [88]. Further advancement in the study of chromatin accessibility was introduced in 2013 by Buenrostro and colleagues with the ATAC-seq method [89]. This assay is a better alternative to the DNAse-seq and FAIRE-seq methods, significantly reducing the amount of sample required (~20,000 cells compared to the millions needed by the other two techniques). ATAC-seq identifies accessible regions of the genome using Tn5, a mutant hyperactive prokaryotic transposase. Tn5 simultaneously fragments and tags with adapters (tagmentation) unprotected regions of the DNA. Thus, Tn5 probes the accessibility of the chromatin at the genomic level [91]. Compared to other methods, ATAC-seq affords better resolution with less starting material and shorter execution time.

The techniques described above have been applied to the study of epigenetics in several contexts, including development, cell differentiation and cancer.

## 5. DamID, Chec-seq and Chic-seq Enzyme-Tethering Strategies Applied to the Study of Chromatin Profiling

**Figure 3:**
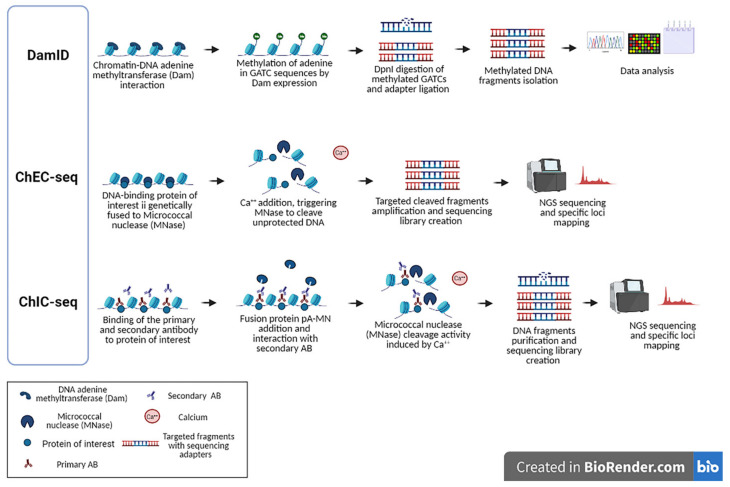
Enzyme-tethering analysis. DamID is used to map the binding sites of DNA and chromatin-binding proteins and consists of fusing DNA methyltransferase to a protein of interest (usually a transcription factor), which methylates adenine in GATC sequences. The genome is digested by DpnI, which cuts only methylated GATCs, that are amplified in PCR assay and subsequently analysed. In ChEC-seq analysis, Micrococcal Nuclease (MNase) cleaves DNA after Ca^2+^ addition, allowing chromatin-associated protein profiling. ChIC-seq procedure is based on binding of the primary antibody (AB) to chromatin-associated protein of interest and on subsequent fusion of protein pA-MN to the secondary AB that produces DNA fragments used for sequencing library creation and data analysis (created with biorender.com).

Enzyme-tethering strategies such as DamID (DNA Adenine Methyltransferase Identification) [92], ChEC (Chromatin Endogenous Cleavage) and ChIC (Chromatin Immunocleavage) [93], are alternative systems to standard ChIP-seq. Their common strategy is to target the DNA of interest directly inside the nucleus of cells rather than after precipitation of DNA–protein complexes (Figure 3).

In 2000, Bas van Steensel and Steven Henikoff introduced a method based on the fusion between the DNA adenine methyltransferase (Dam) enzyme of *Escherichia coli* and a chromatin protein of interest to map the protein contact sites on the DNA. Dam methylates the adenines of adjacent GATC sites in vivo. After DNA extraction, methylated GATC motifs are first enriched using a methylation-sensitive endonuclease (DpnI) that restricts unmethylated sites and then are mapped by sequencing to produce a genome-wide binding profile [92]. Unlike ChIP, DamID does not require antibodies or other affinity reagents. Interestingly, mapping studies using DamID have shown that the nuclear lamina (NL) is mainly associated with inactive chromatin, either directly modulated by epigenetic changes such as histone deacetylation, H3K9me2 or indirectly through secondary targets such as Oct1 in order to repress genes that are located in domains associated with the lamin (LAD) [94]. Thus, DAmID is a method of choice for profiling chromatin proteins associated with nuclear lamina [94] for studying RNA–chromatin interactions, long-range chromosome interactions and chromatin accessibility [95]. Indeed, the study of open chromatin has benefitted from the development of the Chromatin Accessibility Targeted DamID (CATaDA) protocol [95], which has been used extensively in a wide range of model organisms [96,97,98,99].

Subsequently, two complementary methods, ChEC and ChIC, were developed to avoid the bias introduced by the chromatin fragmentation–solubilisation step in ChIP. The two approaches were first used to map the DNA binding sites of CBPs in the baker yeast *Saccharomyces cerevisiae* [93]. In ChEC a chromatin-associated protein of interest is fused to MNase, a calcium-dependent exo-/endonuclease. After gentle permeabilisation, the addition of calcium induces the cleavage activity of MNase and the generation of double-strand breaks around loci bound by the fusion protein [93]. In ChIC, a specific antibody binds the target protein in situ and tethers a protein A-MNase (pA-MNase) fusion. Conveniently, this avoids producing a specific MNase fusion for each protein of interest if efficient antibodies are available [93]. Additionally, after extraction, the total DNA can be size-selected before library preparation, with increased efficiency [93,100]. The proficiency of these methods was demonstrated in yeast by Zentner et al., who mapped the binding of the general transcription factors Reb1, Abf1 and Rap1 [101], and by Grünberg et al., who profiled the Mediator complex, a necessary transcriptional coactivator [102]. In summary, these methods reduce the incidence of background observed in ChIP-based experiments providing high-resolution profiling [103].

More recently, Donczew et al. used these methods to map the specific transcription coactivator’s localisation both in normal and pathological events [104]. Furthermore, Tebbji et al. have used a modified ChEC-seq protocol to unravel the occupancy of the chromatin remodelling complex (SWI/SNF) in *Candida albicans*, which is implicated in fungal fitness and drug resistance [105]. Additionally, scChIC-seq coupled with normal ChIC protocol was able to detect specific histone modifications target sites in the human blood cell clusters [106]. The many advantages obtained in terms of resolution, efficiency and specificity by ChIC and ChEC have highlighted the experimental potential of the enzyme-tethering methods in the elucidation of the epigenetic features and functions of chromatin, paving the way for further improvements in this field. The possibility of studying single-cell chromatin dynamics has opened new frontiers to complementary methods or extensions of ChIP-seq, such as enzyme-tethering, to better understand genomic relationships of protein–DNA interactions (Table 1).

## 6. Emerging Strategies for Efficient Genome-Wide Chromatin Profiling: CUT&RUN and CUT&TAG

Chromatin is crucial in regulating gene expression. Features such as occupancy by transcription factors (TFs), regions of DNA accessibility and histone modifications can vary between cell types, in response to stimuli and in pathological versus healthy conditions.

Thus, a genome-wide understanding of chromatin profiling is needed to characterise pathological changes that occur in disease.

Generally, from its first application, genome-wide chromatin profiling has been performed using the ChIP-seq method [111], but in the last year, some biases have been emerged [76].

Cleavage Under Targets and Release Using Nuclease (CUT&RUN) is an efficient chromatin profiling technique similar to ChIC [100,107]. It is performed on permeabilised nuclei fixed to a solid support. After in situ incubation with a specific antibody against a protein of interest, the addition of pA-MNase and Ca^2+^ results in DNA cleavage. The DNA fragments that have been generated are then used for library preparation. In contrast to other strategies, CUT&RUN has low backgrounds, resulting in a high-throughput genome-wide mapping. It is an attractive strategy that has overcome some methodological limitations such as crosslink and solubilisation issues [100].

In addition to this advantage and in contrast to standard ChIP-seq methodology, CUT&RUN can be carried out in few hours, beginning from only 100 cells for a histone modification profiling and starting from 1000 cells for a transcription factor [100,107].

CUT&RUN has been employed successfully in several model systems. For instance, in *Arabidopsis,* it has been used to generate a genome-wide map of the histone modification H3K27me3 (tri-methylation at lysine 27 of histone 3) in nuclei of the endosperm [112], in the human cancer cell line to profile RNApol II [113] and in a few cell populations as those of early embryos, as reported by Hainer and colleagues, who used some variations to the original technique to profile chromatin proteins occupancy at the single-cell level, rendering this method very adaptable also for cell populations hard to achieve in large numbers [114].

Furthermore, an automated CUT-RUN procedure has been reported (AutoCUT&RUN), providing a high-throughput chromatin profiling of patients for clinical use in the biomedicine field [115].

**Figure 4:**
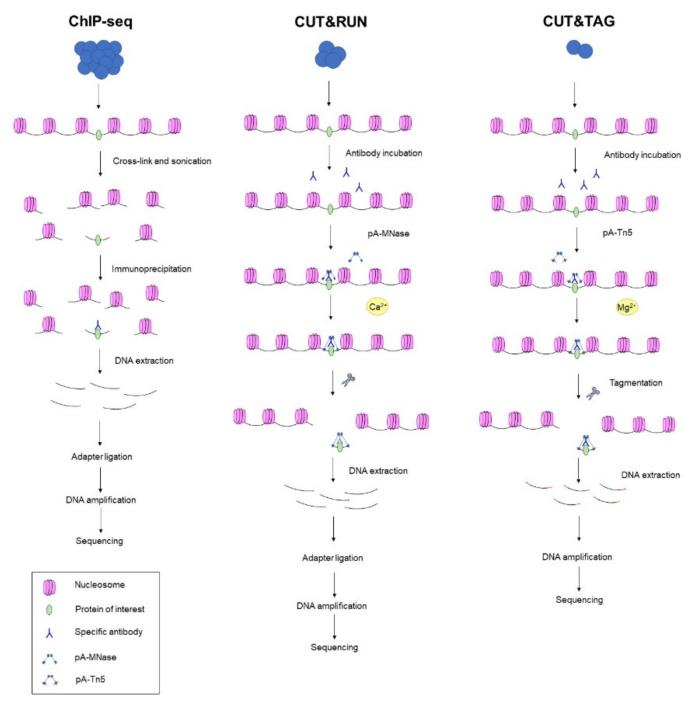
Graphic illustration of the principal technological approaches for epigenetic chromatin profiling. ChIP-seq is a well-used method to profile DNA–protein interactions. In this method, after crosslink and DNA fragmentation, a hybridisation step with a specific antibody against a protein of interest is needed. After DNA extraction, library preparation can be performed. CUT&RUN is an enzyme-tethering strategy that, after in situ incubation with a specific antibody, makes use of a micrococcal nuclease fusion protein (pA-MNase) for DNA fragment generation. CUT&TAG is the most recent methodology introduced for epigenome features profiling. As with CUT&RUN, it begins with an antibody incubation but exploits the transposome Tn5-transposase-protein A (pA-Tn5) fusion protein loaded with sequencing adapters for tagmentation and sequencing.

Derived from CUT&RUN, a modification called Cleavage Under Targets and Tagmentation (CUT &TAG) has emerged in the last few years [108,109,110]. The main improvement is the use of pA-Tn5 loaded with sequencing adapters instead of pA-MNase. This results in the production of DNA fragments ready for sequencing by tagmentation [108,109,110] (Figure 4).

CUT&TAG is quite inexpensive, fast and suitable for single-cell sequencing applications. The main limitation is that it requires access to specific antibodies whose affinity determines the overall efficacy of the reaction [108,109,110].

Compared to ChIP-seq that requires a high number of starting materials, making it not suitable for diagnostic applications, CUT&TAG is a higher-sensitivity technique that can be performed starting from a low number of cells. In addition, CUT&RUN and CUT&TAG have been carried out on unfixed and permeabilised cells, avoiding epitope masking generally associated with the ChIP method, in which DNA and proteins have cross-linked with formaldehyde or UV lights.

CUT&TAG is a low-cost genome-wide chromatin profiling and show crucial advantageous features that allow it to be employed for clinical and diagnostic application in the biomedicine field. In fact, it is not so expensive, can give results in only a day and it does not need a great number of cells, resulting in a single-cell methodology. Indeed, it could be suitable to explore cell-to-cell heterogeneity, a hallmark of cancer cells.

Despite being a very new technique, there are several publications describing CUT&TAG applications. For instance, the genomic profiling of native R loops, which plays a pivotal function in many biological processes with consequences in human disease [116]. Yu et al. have employed the H3K27Ac CUT&Tag protocol to investigate BMP-dependent active enhancers during osteoblast differentiation [117].

Bartosovic et al. used CUT&TAG to profile histone modifications and transcription factors occupancy at the single-cell level in the mouse brain [118].

In addition to mammalian cells, the CUT&TAG technique has been recently used for epigenetic profiling in cotton cells, leading to improvement in plant epigenetic research and giving the opportunity to widely employ this methodology in other model systems [119,120].

CUT&TAG can be regarded as an ideal method to investigate cellular heterogeneity in physiological conditions and in human disorders such as in a patient tumour mass.

In particular, we have already mentioned that the cancer cells population is characterised by a high heterogeneity that drives crucial issues in diagnosis and mostly in therapy and drug resistance. For this reason, single-cell omics techniques appear as optimal methodologies to investigate alterations at the single-cell level in cancer mass.

Of note, Wu and colleagues developed the CUT&TAG technique for single-cell chromatin profile of brain tumour cells from an autopsy of a patient before and after cancer therapy, giving the opportunity to explore tumour heterogeneity [121].

Therefore, the improvement of methodologies helps us to understand better physiological epigenetic mechanisms, clarifying alterations involved in diseases.

The centromere is a typical example of epigenetic regulation. Indeed, it is an epigenetically regulated chromosome structure critical for fidelity in cell divisions. Alterations in centromere regulation can induce aberrant mitosis and chromosome instability observed in cancer and many pathologies.

Therefore, it is crucial to gain insight into mechanisms that control epigenetic regulation of the centromere to control aneuploidy in cancer cells since several evidences show a correlation between centromere dysfunction and neoplasia [122,123].

These emerging single-cell techniques could be used in the next few years to elucidate more in-detail epigenetic regulation of the centromere, contributing to understanding pathological mechanisms in diseases.

Finally, these enzyme-tethering techniques, and in particular CUT&TAG, are efficient methods for epigenetic factors profiling in a high-resolution manner and low background, using low cell numbers or single cells, opening the way to epigenetic studies in any field of biological research.

## 7. Epigenetics Approaches to Explore the Architecture of Centromeric Chromatin

Centromeres are regions of the chromatin that are crucial for the faithful segregation of chromosomes during mitosis. They are the site of assembly of the kinetochore complex, a proteinaceous structure that binds to the microtubules of the mitotic spindle driving chromosome segregation. Their disfunction can lead to genome instability or to an aberrant cell cycle resulting in tumorigenesis [124].

Centromeres are characterised by repetitive DNA sequences [125,126] and retroelements [127,128,129,130]. However, it seems that specific DNA sequences are neither necessary nor sufficient for centromere specification. Instead, centromeres are defined epigenetically by the presence of the centromere-specific histone H3 variant, centromeric protein A (CENP-A). This is a highly conserved protein found in many organisms [131,132,133].

A specific type of centromeric chromatin, named ‘centrochromatin’ is found in addition to CENP-A. This is characterised by a specific pattern of histone modifications that is crucial for CENP-A recruitment and centromere function [134,135,136]. Using human artificial chromosome (HAC) it has been possible to identify histone modifications such as H3K9me, H3K4me and H3K27ac and to show that centromeric transcription is required for centromere function. Additionally, tethering repressive and heterochromatic factors such as Suv39h1 and HP1 or enzymes that demethylate H3K4, a marker of active chromatin, causes loss of centromeric transcription and inactivation of centromere function [136,137,138].

A correct balance between specific epigenetic modifications is required to promote the loading of CENP-A to centromeric regions and to prevent the spreading of heterochromatin, avoiding centromere and kinetochore inactivation [136].

ChIP experiments have enabled most studies on the epigenetic regulation of the centromere. Notably, it has been difficult to map histone modifications on centromeric repetitive DNA sequences employing ChIP-seq methodologies [139] as they require a large number of cells and they do not consider a heterogeneous population of cells.

As with other histones, CENP-A is also modified by post-translational modifications, including phosphorylation, methylation, acetylation and ubiquitination, which are critical for centromere specification [140,141,142,143].

The characterisation of CENP-A post-translational modifications can have a pivotal role in cancer research. Because they may lead to chromosome segregation defects and chromosomal instability, they could be helpful diagnostic markers or therapeutic targets [144]. Moreover, the complex relationship between CENP-A overexpression and defects in chromosomal stability needs further investigation [145].

Employing emerging techniques, it would be interesting to analyse CENP-A modifications at the single-cell level in cancer cells to better evaluate their contribution to cancer initiation and progression, which could become a helpful diagnostic marker or therapeutic target.

Thus, investigating specific histone modifications, including CENP-A modifications, could be an exciting topic for future perspectives in order to further explore epigenetic mechanisms in centromere regulation.

Indeed, innovative genome-wide approaches to study epigenetic features as chromatin modifications are recently developed, revealing new aspects of biology. The application of these techniques has greatly advanced our knowledge of epigenetic processes and how they are regulated and could be used for centromere regulation exploration.

In the last decade, *C. elegans* centromeres have been analysed using ChIP-on-chip with CENP-A antibodies. These investigations have confirmed CENP-A presence along the chromosome arms. In addition, a link has emerged between transcription and CENP-A deposition in nematodes. In fact, CENP-A is found in chromosome regions that are not transcribed in the germline and in the first stages of embryonic development [146].

Usually, centromeres occupy specific chromosome regions, but sporadicallym they shift to ectopic sites driving neocentromere formation. Neocentromeres have been identified in different organisms. Generally, they are formed following a chromosomal rearrangement to stabilise an acentric chromosome. Such events are still poorly understood, although some models have been proposed [147,148,149,150,151,152].

Different methodologies provide an opportunity to investigate such rare events. For instance, ChIP-seq has been instrumental in exploring the epigenetic environment surrounding neocentromeres. Shang et al. have described a chromosome-engineering system to experimentally generate neocentromere in chicken DT40 cells. Using a specific CENP-A antibody and ChIP-seq, they demonstrated the presence of CENP-A in ectopic centromeres and clarified the epigenetic features occurring in those regions [153].

Recently, Pineda and colleagues described for the first time a system to characterise a ‘spontaneous’ neocentromere in human culture cells, opening a new era for centromere research.

In this work, they could observe early driven events and subsequently step-by-step neocentromere maturation in cellular generations. First, using CENP-A immunoprecipitation followed by long-read DNA sequencing, they identify genomic features of neocentromere confirming the epigenetic nature of the event supposed previously [154,155].

In some cases, new centromeres have been observed in patients with developmental disorders during karyotyping analysis [147] and in human cancer such as lipomatous tumours and acute myeloid leukaemia (AML) [150].

Indeed, chromosomal aberrations are a common feature in cancer pathogenesis, and genomic anomalies can contribute to neoplastic progression and metastasis [156,157].

CENP-A is often overexpressed in some type of tumors, and for this reason, its detection could have diagnostic and prognostic values [25,26,27]. In fruit flies, overexpression of CENP-A results in new centromere formation [158,159,160], suggesting that CENP-A is sufficient to initiate the assembly of de novo centromeres. Evidence from human cells shows that CENP-A incorporation in ectopic sites leads to the recruitment of other centromeric components. In cancer cells, CENP-A overexpression can be detrimental, leading to neocentromere formation, chromosomal instability and growth advantage, increasing tumour malignancy [28].

For instance, CENP-A deposition in ectopic sites can generate a neocentromere, leading to a dicentric chromosome and chromosome rearrangements. Moreover, CENP-A can change endogenous transcription and alter gene expression.

Recent studies have shown that CENP-A plays a protective role in centromere integrity, preventing centromeric satellite DNA from rearrangements. This suggests that centromere aberrations in cancer cells could be associated with CENP-A dysfunction [161].

Chromosomal instability is a hallmark of human cancer. Tumorigenesis is a stepwise process that leads to heterogeneity in cell populations. Therefore, it may be helpful to monitor cell diversity over time to characterise cancer evolution and to inform therapeutic strategies.

Recently, Bolhaqueiro et al. reported a system to detect chromosomal instability in human colorectal tumour cells during cell division using microscopy. In addition, they repeatedly performed sc-Karyo-seq, a single-cell DNA sequencing technique, to detect chromosomal changes across cell divisions, revealing karyotype heterogeneity [24].

To study centromeric chromatin in native conditions, Thakur and Henikoff have employed CUT & RUN combined with salt fractionation (CUT&RUN Salt). CUT and RUN, as previously described, is a powerful method for chromatin profiling. The authors demonstrated that even slight differences in centromeric satellite sequences could cause changes in the architecture of the chromatin and in the binding of proteins to the centromere [34,162].

Using innovative single-cell techniques such as CUT&TAG, it would be interesting to study in detail if CENP-A islands in non-canonical regions of the genome differ from one cell type to another and investigate whether CENP-A occupancy in ectopic sites occurs at random or if it localises in defined genome regions following some sequence-specificity.

Hence, the CUT&TAG methodology could be useful to investigate CENP-A attendance in the genome at the single-cell level.

Finally, we have shown how ChIP-seq and other omics techniques have been helpful to investigate epigenetic features on centromeric chromatin such as CENP-A distribution and histone post-translation modifications in endogenous centromere and in ectopic centromeres. Indeed, these techniques have contributed to clarifying epigenetic mechanisms that drive neocentromere formation and characterising genomic features of de novo centromeres.

In light of this, we can speculate that innovative technological approaches could be suitable to rather explore epigenetic involvement in centromere specification and useful for epigenetic studies in clinical research and biomedicine.

## 8. Conclusions

Epigenetic modifications are what determine the specialisation of a cell, and for this reason, epigenetic mechanisms impinge on fundamental biological phenomena such as gene expression and chromosome integrity.

Alterations of epigenetic processes have been associated with multiple diseases, for example, behaviour plasticity, memory, cancer, autoimmunity, addiction as well as neurodegenerative and psychological disorders. Thus, epigenetic studies have become crucial for understanding physiological and pathological conditions. Dissecting the regulation of epigenetic pathways and establishing their connection with environmental changes is technically challenging, but novel approaches have become available in the last few years.

The most widely used technique for chromatin profiling is ChIP-seq which has been instrumental in describing chromatin features such as protein binding sites, transcription factors occupancy and correlated histone modifications.

However, biases have emerged, especially when applying the technique to smaller and smaller samples, leading researchers to develop other methodological approaches to bypass ChIP-seq limitations.

Technically, ChIP methods are afflicted by high background due to cross-linking, poor solubility of DNA–proteins complexes and poor affinity of some antibodies. Thus, traditional ChIP assays can provide a snapshot of DNA–protein interactions at any one time but are quite poor at providing kinetic information.

A powerful counterpart to ChIP comes from the strategy of using tethered enzymes. ChIC-seq and ChEC-seq allow the investigation of protein–DNA interactions over time, providing an independent means by which to compare the results of ChIP-seq and discover new insights on genomic regulation.

Finally, CUT&RUN and CUT&TAG allow producing high-quality chromatin profiling from low cell numbers using a relatively quick and easy protocol.

Indeed, we reported how single-cell methodologies play a key role in dissecting epigenetic mechanisms underlying pathologies and chromosomal instability in cancer cells.

Recent enhancements in technical methods have offered opportunities to elucidate the epigenetic environment in human disorders. Other single-cell methodologies need to be developed in the future for personalised medicine.

## Figures and Tables

**Table 1:** Main features of the epigenetic techniques discussed in this review.

Method	Typical Cell Input (Cells)	Advantages	Disadvantages	Reference
ChIP-seq	≥5 × 10^5^	Methodology for genome-wide mapping of protein-DNA interactions Availability of database sequences	Sonication and crosslinking-related interferences Dependent on antibody efficiency	[69]
DNAse-seq	≥10^6^	Identification of regulatory regions	High cell input typically required Specificity for DNA regions not wrapped on histones	[86]
FAIRE-seq	≥10^5^	Simple and rapid protocol Crosslinking takes place directly between DNA and genomic portions nucleosomes-free	Low signal-to-noise ratio Dependent on correct crosslink efficiency	[88]
MNAse-seq	≥10^6^	Map nucleosome and TF information Chromatin processing by Micrococcal Nuclease (MNAse) action	High cell input typically required	[89]
ATAC-seq	≥5 × 10^4^	Low cell input requirement Fast and easy protocol Native conditions	Correct combination in adapter-transposase mixture needs to be optimized Distance between adapter sites must be checked for library amplification	[92]
Dam-ID	≥10^4^	No antibody, sonication and crosslink-dependent Low base-pair resolution GATC motifs-dependence of Dam action	Use of fusion protein Dependent on GATC sequences	[93]
CHEC-seq	≥5 × 10^7^	No antibody, sonication and Crosslink-dependent Inducible	Chromatin-MN fusion protein expression	[85]
CUT&RUN	≥10^5^	Very low cellular input Fast and easy protocol High-throughput genome-wide mapping Native conditions and inducible system	Quality antibody-dependence	[100,107]
CUT&TAG	≥5 × 10^5^	Low-cost and very fast technique High-throughput genome wide mapping Low background and high resolution pA-Tn5 loaded with sequencing adapters Native conditions and inducible system	Quality antibody-dependence	[108,109,110]

## Data Availability

Not applicable.
